# Supplementary material for: Thermal and electrical signatures of a hydrodynamic electron fluid in tungsten diphosphide
Source: Nat Commun. 2018 Oct 5;9:4093. doi: 10.1038/s41467-018-06688-y (PMC6173759; doi:10.1038/s41467-018-06688-y)
Supplement: Supplementary file 1 — Supplementary Information [file 41467_2018_6688_MOESM1_ESM.pdf]

Supplementary Information

Thermal and electrical signatures of a hydrodynamic electron fluid in tungsten diphosphide

J. Gooth, *et al.*

Supplementary Note 1: Estimation of band structure parameters from magnetoelectrical electrical transport on the bulk samples

Magnetic field-dependent electrical resistance measurements on the WP_2 bulk sample, revealed an enormous, highly anisotropic magneto-resistance (MR)[1]. The anisotropy has been attributed to orbital motions of charge carriers on the anisotropic Fermi surfaces, driven by the Lorentz force. Such effect has previously been reported[2] in $PdCoO_2$, which becomes hydrodynamic at low temperatures[3]. The anisotropy of the Fermi surface manifests itself in the Fermi velocity v_F, which is directly related to the mean free path l_{mr} in the different crystal planes perpendicular to the magnetic field. The MR is, therefore, maximum when the field is applied along the *b*-axis of the crystal and is decreased by 2.5 orders of magnitudes when the field is rotated to the *c*-axis. Consequently, the l_{mr} in the *a*-*c* plane is 250-times larger than in the *a*-*b* plane.

From the Hall (Supplementary Fig. 1 (a)) measurements, we obtain the temperature (T)-dependent average carrier concentration $n = (d\rho_{xy}/dB\ e)^{-1}$ above 30 K from a single band model. Below 30 K, the Hall resistivity becomes non-linear, indicating the contributions of multiple bands to the transport. At these low temperatures, four frequencies f were determined from Shubnikov-de Haas oscillations when the magnetic field is applied along the *b*-axis, of which two are hole-pockets α and β (f_α = 1460 T and f_β = 1950 T) and two are electron-pockets γ and δ (f_γ = 2650 T and f_δ = 3790 T) [1]. These pockets were identified using the modified Becke-Johnson method. We calculated the k-space areas of the extremal cross-sections of the Fermi surfaces perpendicular to a magnetic field that is applied along the *b*-axis of the crystal and found four frequencies at around 1300 T, 1900 T, 2800 T and 3900 T, which reflect the experimentally obtained frequencies well. We have recently determined the effective masses of the hole-pockets from the Shubnikov de-Haas oscillations as $m_\alpha = 1.67\ m_0$ and $m_\beta = 1.89\ m_0$. To account for all bands at the Fermi level, we additionally calculated the effective masses of

the electron-pockets as $m_{\gamma} = 0.87\ m_0$ and $m_{\delta} = 0.99\ m_0$, where m_0 denotes the free electron mass (Supplementary Fig. 2).

From the Onsager relation, we determine the size of the Fermi surface cross sections $A_{Fi} = 2\pi^2 f_i / \phi_0$ with ϕ_0 as the magnetic flux quantum and the sub-index i reflecting α, β, γ and δ. Applying the standard circular approximation, we obtain momentum-vectors $k_{Fi} = (A_{Fi}/\pi)^{1/2}$ of $k_{F\alpha} = 2.11 \times 10^9$ m^{-1}, $k_{F\beta} = 2.40 \times 10^9$ m^{-1}, $k_{F\gamma} = 2.84 \times 10^9$ m^{-1} and $k_{F\delta} = 3.39 \times 10^9$ m^{-1}. Fermi liquid theory in the limit of zero temperature results in the total carrier concentration of $n = \sum n_i = 2.9 \times 10^{21}$ cm^{-3}, where $n_i = k_{Fi}^3/(3\pi^2)$. Given the carrier concentrations for the whole temperature range investigated (Supplementary Fig. 1 (b)), we now calculate the average mobility $\mu = (\rho e n)^{-1}$ of the WP_2 bulk sample (Supplementary Fig. 1 (c)). The corresponding Fermi velocities $v_{Fi} = \hbar k_{Fi} / m_i$ are $v_{F\alpha} = 1.46 \times 10^5$ ms^{-1}, $v_{F\beta} = 1.47 \times 10^5$ ms^{-1}, $v_{F\gamma} = 3.78 \times 10^5$ ms^{-1} and $v_{F\delta} = 3.97 \times 10^5$ ms^{-1}. To account for all band contributions in the electrical transport, we calculated the harmonic mean giving the average effective mass $m^* = 4(1/m_{\alpha} + 1/m_{\beta} + 1/m_{\gamma} + 1/m_{\delta})^{-1} = 1.22\ m_0$ and the mean Fermi velocity $v_F = (\sum v_{Fi})/4 = 2.67 \times 10^5$ ms^{-1}. The l_{mr} in the a-c plane is then determined as a function of temperature from the T-dependent mobility $\mu = e v_F l_{mr} / m^*$.

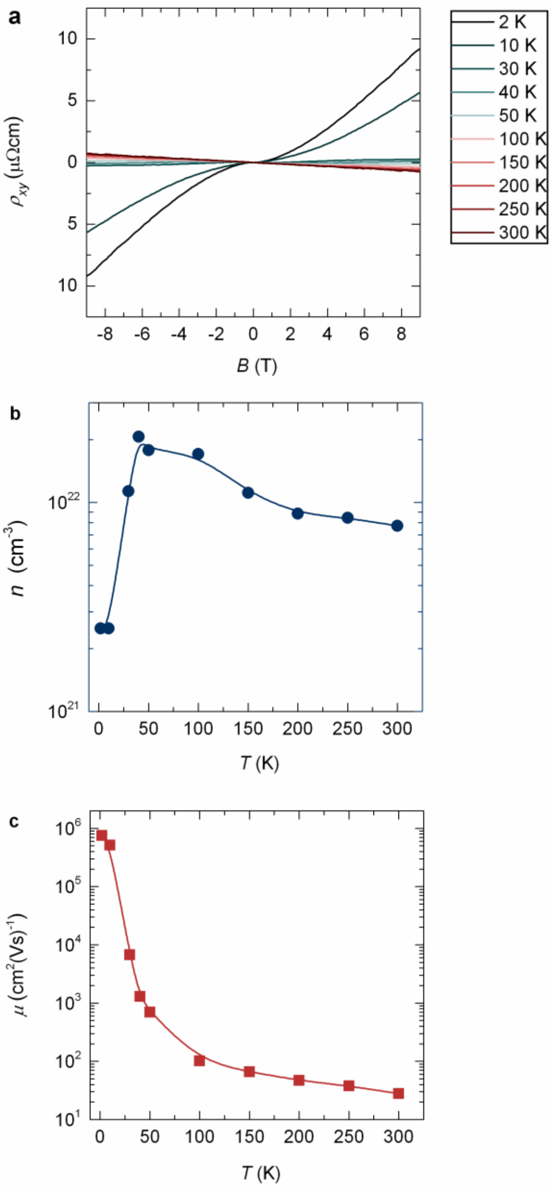

Supplementary Figure 1: Hall measurements on the bulk samples. (**a**) Hall resistivity ρ_{xy} as a function of magnetic field B at various temperatures. (**b**) Extracted total carrier concentration n and (**c**) average mobility μ as a function of temperature T. n is decreasing with increasing temperature due to electron-hole compensation.

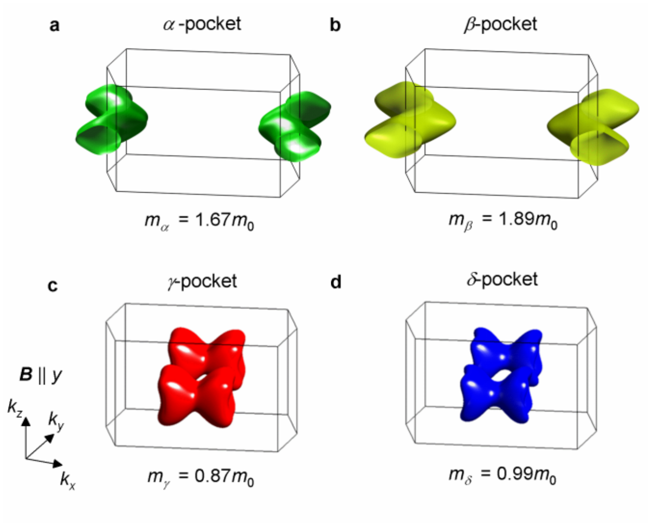

Supplementary Figure 2: Fermi surface and effective mass calculations of (**a**) the α-pocket, (**b**) the β-pocket, (**c**) the γ-pocket and (**d**) the δ-pocket, for a magnetic field B applied along the b-axis of the WP_2 crystal (here y-axis).

Supplementary Note 2: Interface resistance at the metal/semimetal junction

The interface resistance is an important issue, dealing with low sample electrical resistances in the range of mΩ. We therefore have carried out three independent cross-checks to evaluate interface resistance in our devices. All three methods show independently that the interface resistance at the metal/semimetal junction is negligible small in our experiment, despite the low sample resistance:

A common method to evaluate the contact resistance is to compare four 4- and 2-point (or quasi-4-point) measurements. Quasi-4-terminal (red) and actual 4-terminal (blue) resistivity of the 9 μm-wide WP_2 ribbon as a function of temperature are shown in Supplementary Fig. 3. The deviation between the two curves is below 1 % at 300 K and rises to 10 % at 4 K. We note, however, that this enhancement at low temperature is within the measurement error, due to the low resistivity in this temperature range. The 9 μm-wide has the lowest resistance and should therefore be most sensitive to the interface resistance.

Further, we have used a Focused Ion Beam to cut out four-terminal devices. Two devices of 0.8 μm and 3.5 μm width have been fabricated (Supplementary Fig. 4). The resistivity data points obtained from these devices fit in perfectly to the width-dependent series.

Next, we have checked the residual resistivity ρ_0 of the width-dependent resistivity, fitting the data by $\rho = \rho_0 + \rho_1 w^{\beta}$. As shown exemplarily in Supplementary Fig. 4 for the 4 K data, we find ρ_0 matching the resistivity values of the bulk samples measured in Kumar et al.[1]. Explicitly, $\rho_0(4\ \text{K}) = (4.1 \pm 0.4)$ nΩcm.

An important cross-check that the evaluated ρ_0 itself does not introduce the width dependence is to determine ρ_0 from the magnetic field-dependent resistivity data of each width independently. We therefore fit each magnetoresistance curve individually (Supplementary Fig. 5) by the $\rho = \rho_0 + \rho_{1,a} w^{\beta}/(1+(\rho_{1,b}B)^2)$, where B is the magnetic field and β is extracted from the

width-dependent analysis above. As shown in Supplementary Fig. 6, all individual curves result in a ρ_0(4 K) of around 4, in full agreement with the previous analysis.

Therefore, we conclude that within our measurement precision, the metal/semimetal interface perfectly transmits charge carriers and does not represent a noticeable resistance. This could indicate a different mechanism for resistances at hydrodynamic/normal metal electron interfaces.

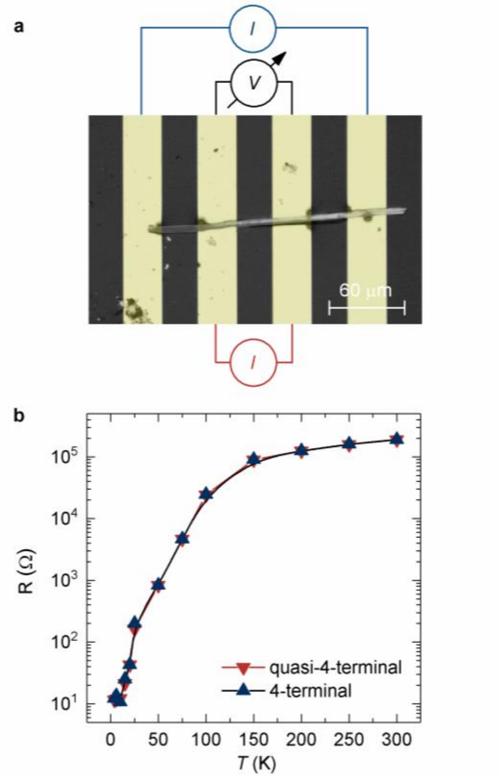

Supplementary Figure 3: Four-point versus quasi-four-point measurements. **(a)** Sketch of the transport configuration. The bias current *I* is employed along the *a*-axis of the crystal in a quasi-4-terminal configuration at the inner contacts (red) and in an actual 4-terminal configuration (blue) at the outer contacts. The voltage response *V* is measured at the inner contacts. (**b**) Quasi-4-terminal (red rectangles) and actual 4-terminal (blue rectangles) resistivity of the 9 μm-wide WP_2 ribbon as a function of temperature. The deviation between the two curves is below 1 % at 300 K and rises to 10 % at 4 K. We note, however, that this enhancement at low temperature is within the measurement error, due to the low resistivity in this temperature range.

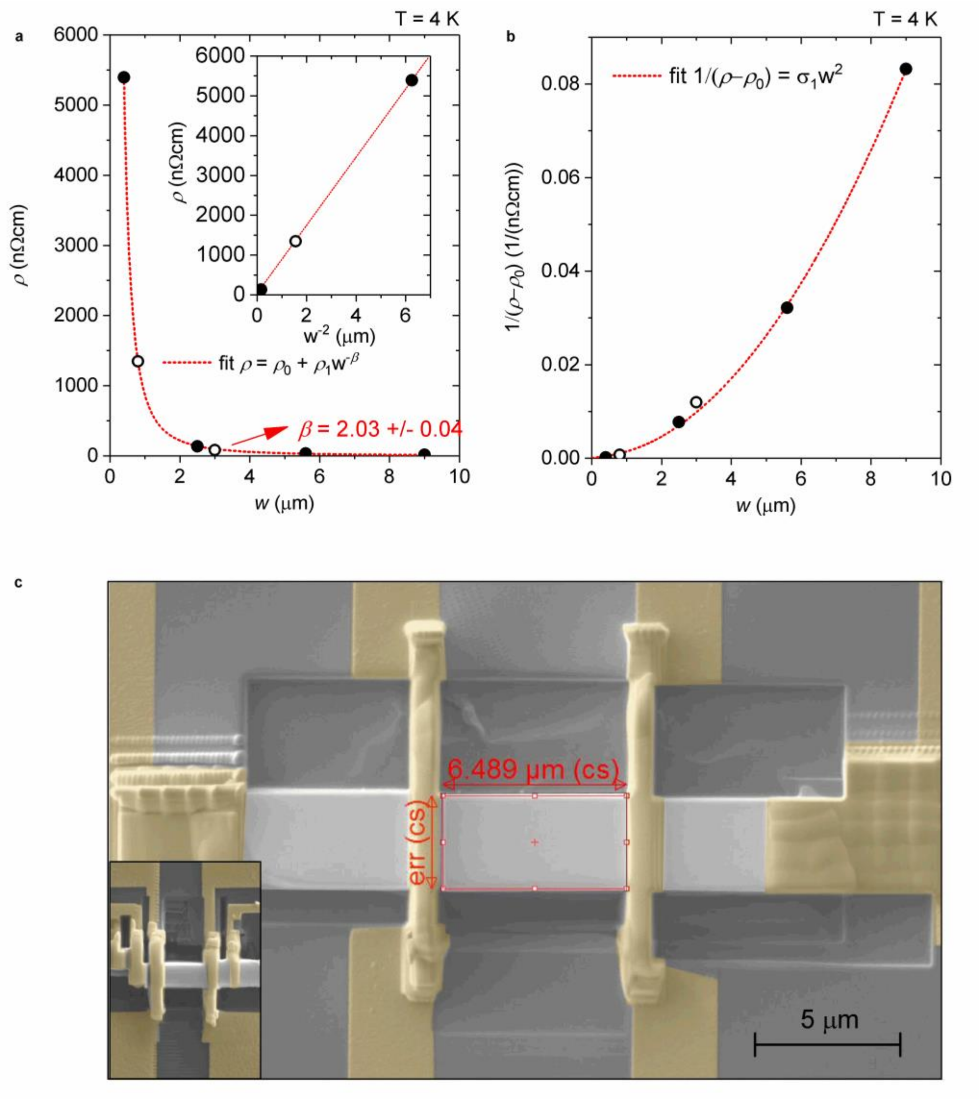

Supplementary Figure 4: Width-dependent electrical resistivity fits. (**a**) Measurements data (black dots) of the resistivity ρ versus width w at 4 K, fitted (red dashed line) by $\rho = \rho_0 + \rho_1 w^{\beta}$. We extract the power of $\beta = 1.96 \pm 0.03$ and a residual resistivity of ρ_0(4 K) = 4.1 ±0.4 nΩcm. The filled symbols denote quasi-four terminal measurements, the open symbols denote four-terminal measurements. The inset shows the measurement data and fit of ρ versus w^{-2}. (**b**) $1/(\rho\text{-}\rho_0)$ versus w, showing the w^2-dpendence of the viscous conductance at 4 K. (**c**) Scanning electron microscope image of the four-terminal devices, cut by a Focused Ion Beam. The scale bar counts for both images.

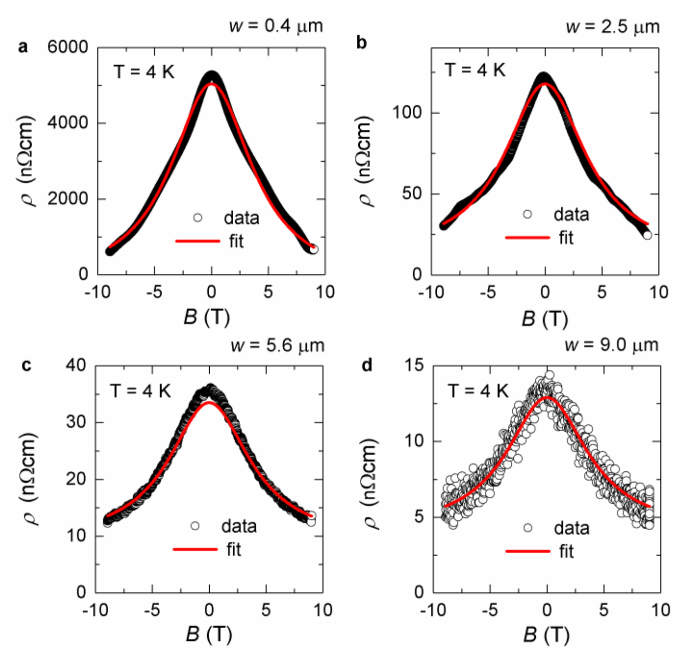

Supplementary Figure 5: Magneto-resistivity at 4 K of (**a**) the 0.4 μm-wide sample, (**b**) the 2.5 μm-wide sample, (**c**) the 5.6 μm-wide sample and the (**d**) the 9.0 μm-wide sample. The black open symbols denote the measurements data and the red lines are best fits of $\rho = \rho_0 + \rho_{1,a} w^{\beta}/(1+(\rho_{1,b}B)^2)$, from which the residual resistivity ρ_0 can be extracted for each width individually.

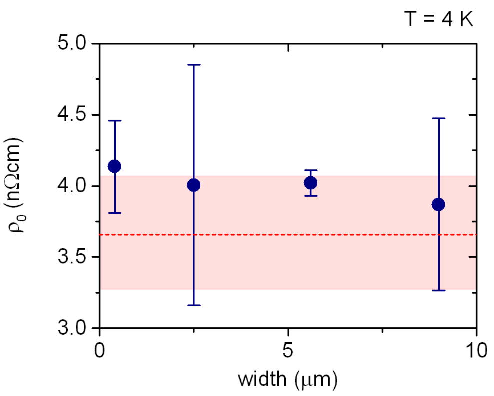

Supplementary Figure 6: Comparison of the residual resistivity ρ_0 at 4 K, extracted from the zero-field width-dependent electrical resistivity (red dashed line, the light red area denotes the error from fit) shown in Supplementary Fig. 4 and from the magneto-resistivity (blue dots, error bars denote the errors from the fits) shown in Supplementary Fig. 5. The differently extracted ρ_0 are similar, demonstrating the consistency of our results and analysis.

Supplementary Note 3: Exponent of the functional dependence of ρ on w

Because momentum-relaxation processes are always present in the bulk of the investigated material system, momentum can only be quasi-conserved. Therefore, the measured resistivity ρ always contains a width-independent Drude offset ρ_0 from the remaining bulk scattering and a width-dependent power-law component $\rho_1 w^\beta$. To analyse the power law component in more detail, we have subtracted ρ_0 from ρ at all temperatures fitting the experimental data with $\rho = \rho_0 + \rho_1 w^\beta$. The obtained exponents β as a function of temperature are subsequently cross-checked by a logarithmic analysis of $\rho - \rho_0 = \rho_1 w^\beta$ (Supplementary Fig. 7). $\log(\rho - \rho_0) = \log(\rho_1 w^\beta) = \log(\rho_1) + \beta \log(w)$ is a linear equation in $\log(w)$ with slope β, which can be obtained from the slope of linear fits to a log-log plot of ρ vs. w. The determined β are in excellent agreement with each other. Employing the hydrodynamic model for $\beta = 2$, the viscosity can be calculated from $\rho_1 = m^*/(e^2 n) \cdot 12\eta$.

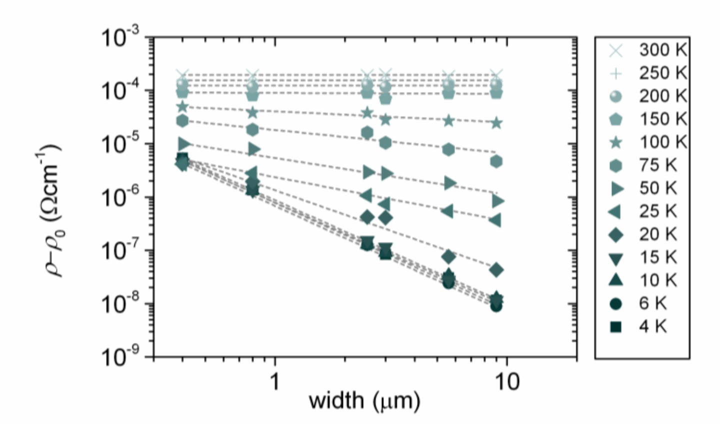

Supplementary Figure 7: Extraction of the exponent of the functional dependence of $\rho - \rho_0$ on w at various temperatures. The slope of the linear fits (dashed lines) from the experimental log-log data (symbols) is plotted in Fig. 1 (d).

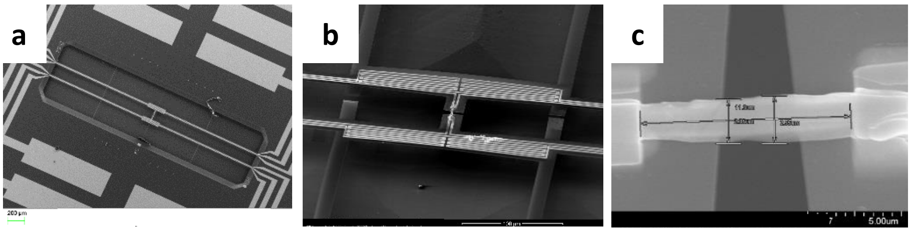

Supplementary Figure 8: The MEMS platform for thermal measurements. (a) Scanning electron micrograph of measurement device similar to the one used in this study prior to mounting the sample. The 1.2 mm-long MEMS beams are etched out of silicon nitride and carry three gold lines each. (**b**) Enlarged area of the measurement device with mounted WP_2 sample. The stripes for stabilization the structure during fabrication (connecting the two heater areas) were cut using a focused ion beam. (**c)** Enlarged image of sample piece. Note that the WP_2 sample is in mechanical contact only with the gold and the deposited Pt lines, but not with the nitride membrane that forms a V-shaped gap.

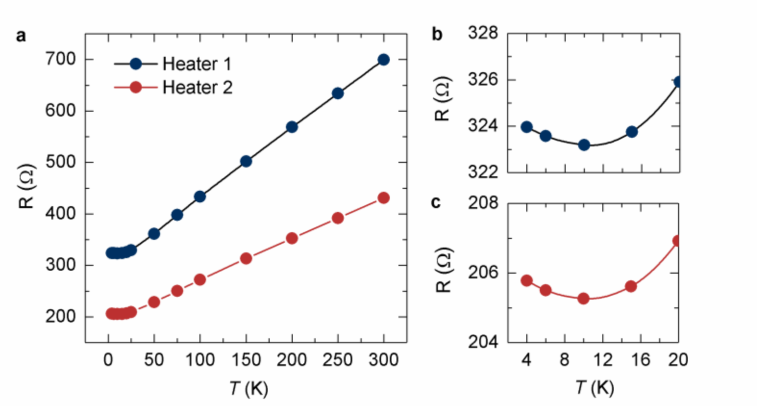

Supplementary Figure 9: Temperature calibration plot of the two heater/sensors of the measurement device. (a) The plot shows the data of heater 1 and heater 2 (extrapolated resistance to zero drive current) as dots and the calibration fits as lines versus cryostat temperature. (**b**) and (**c**) are zoom-ins at low temperatures for heater 1 and 2, respectively.

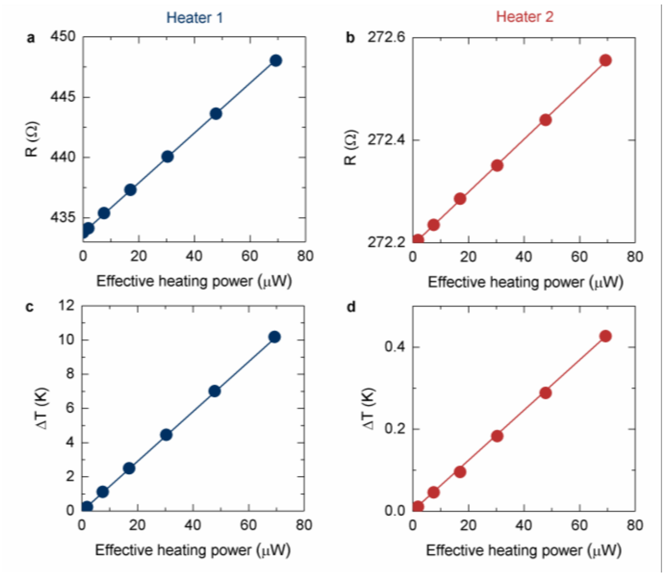

Supplementary Figure 10: Example of the analysis of the data used to calculate the thermal conductance of the WP_2 sample. Here data for a cryostat temperature of 100 K is shown. Resistance of heater (blue) (**a**) and sensor (red) (**b**) for the given heating and sensing currents. Temperature rise above 100 K for heater (**c**) and sensor (**d**). The symbols denote measurement data, the lines linear fits.

Supplementary Note 4: Considerations of Thermal Contact Resistance

To minimize the influence of thermal contact resistance, we fabricated electrical contacts to the sample with a contact area to the metal of 16 μm^2. For the phonon contribution of the thermal boundary conductance we expect values of the order of 10^{-8} to 10^{-7} Km^2W^{-1}. Therefore, the thermal resistance of the contacts will be on the order of 10^4 – 10^5 KW^{-1}, which is small compared to the measured overall resistance of 1 to 4×10^6 KW^{-1}. Moreover, if we include the electron contribution to thermal conductance into this consideration, we expect the difference will be an order of magnitude further apart. Note, that although WP_2 is classified as a semimetal, we have an additional band at the Fermi energy contributing to the sample carrier density. The large contact size, however, increases the systematic error in the sample dimension length used in the analysis.

Supplementary Note 5: Additional magneto-hydrodynamic analysis

Employing the hydrodynamic model for $\beta = 2$, ρ_1 can be expresses as $\rho_1(B) = m^*/(e^2n)\cdot 12\,\eta(B)w^{-2}$, with $\eta(B) = \eta_0/(1+(2\,\tau_{er}\,\omega_c)^2)$. From this expression, we can extract τ_{er} as a function of temperature from the data in Supplementary Fig. 11 and calculate the viscosity $\eta = v_F^2\,\tau_{er}/4$ as a function of temperature. As shown in Supplementary Fig. 12, the viscosities extracted from the width-dependent zero field data (Supplementary Fig. 7) and extracted from the field-dependent data in of Supplementary Fig. 11 are in excellent agreement. This agreement between the viscosities extracted from independent dependencies is an important cross-check of our interpretation and shows the consistency of our results.

With $v_F = l_{er}/\,\tau_{er}$ from the bulk analysis above, we can extract the momentum conserving length l_e for each width independently and compare it to the momentum relaxing mean free oath from the Hall analysis. As exemplarily shown in Supplementary Fig. 13 at 4 K, we find an excellent agreement between the mean free paths of the different widths. The obtained size-regime validates the application of the hydrodynamic model for the data where $\beta = 2$.

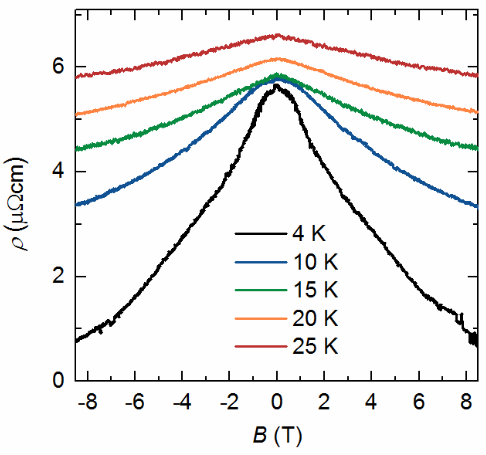

Supplementary Figure 11: Electrical resistivity ρ of the 2.5 μm-wide beam as a function of magnetic field B at various temperatures.

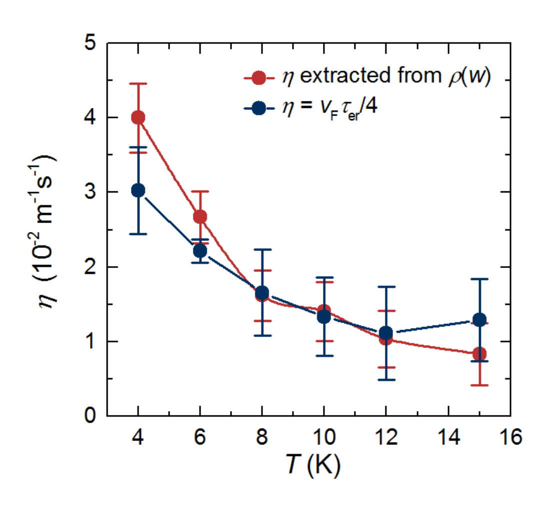

Supplementary Figure 12: Kinematic shear viscosity η versus temperature. The data is extracted from the functional dependence $\rho \sim w^{-2}$ (Supplementary Fig. 11) (red dots) and from the relaxation times τ_{er} as $\eta = v_F^2 \tau_{er}/4$ (blue dots). The lines are guides to the eye. The error bars denote the errors of the accompanied fits.

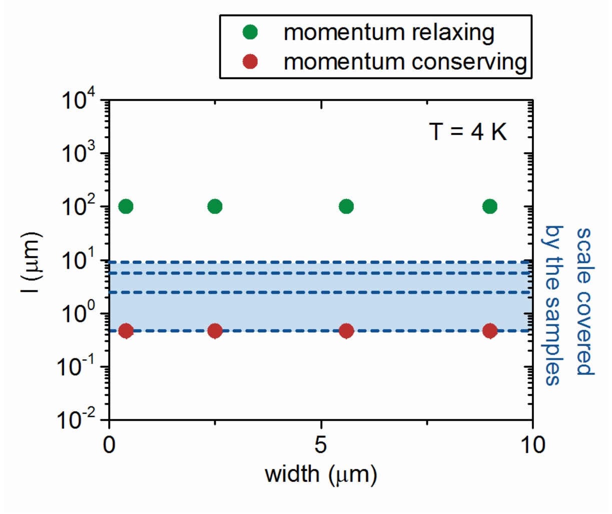

Supplementary Figure 13: Momentum-relaxing (green dots) and momentum-conserving (red dots) scattering length at 4 K, obtained from individual fits of the data in Supplementary Fig. 5 by the Navier-Stokes flow model. The blue dashed lines denote the width of the individual measured samples. The light blue area shows the investigated size-range that lies well within the hydrodynamic regime at the boundary to ballistic crossover. However, we do not observe signatures for ballistic transport in our experiments.

Supplementary Note 6: Additional key quantities

To guide future interpretations of the data, we have calculated additional key quantities that are connected to the strength of the electron-electron interaction. In Supplementary Fig. 15 and Supplementary Fig. 16, we plot the ratio of dynamic viscosity and number density (η_D/n) in units of $\hbar$ as a function of temperature and magnetic field, respectively. η_D/n is directly related to the momentum diffusivity.

Also, $l_{mr}k_F$ is a useful dimensionless quantity that characterizes the strength of interactions. $l_{mr}k_F$ > 1 clearly supports the existence of quasiparticles in WP_2, in agreement with the observed Shubnikov-de Haas oscillations in the bulk samples[1].

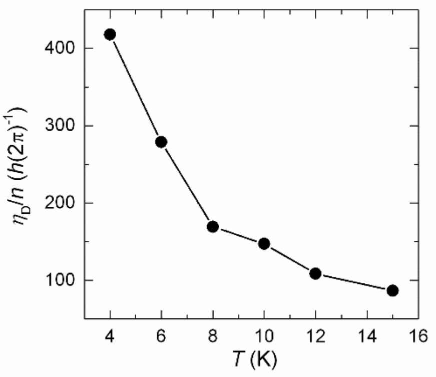

Supplementary Figure 14: Ratio of dynamic viscosity and number density (η_D/n) in units of $\hbar$ as a function of temperature.

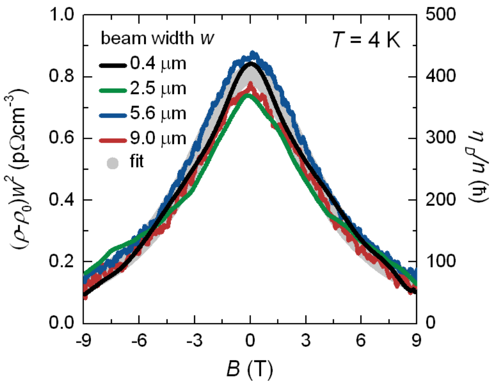

Supplementary Figure 15: Ratio of dynamic viscosity and number density (η_D/n) in units of $\hbar$ as a function of magnetic field at 4 K (right axis). Left axis: $(\rho-\rho_0)w^2$.

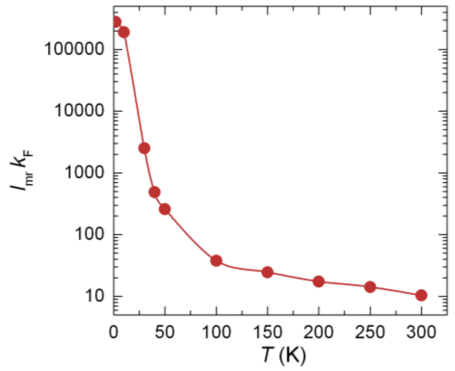

Supplementary Figure 16: $l_{mr}k_F$ as a function of temperature. $l_{mr}k_F > 1$ clearly supports the existence of quasiparticles in WP_2. This, however, does not directly imply anything about correlations.

Supplementary References

1. N.Kumar *et al.*,Extremely high magnetoresistance and conductivity in the type-II Weyl semimetals WP_2 and MoP_2. *Nat. Commun.* **8**, 1642 (2017).

2. Takatsu, H. *et al.* Extremely large magnetoresistance in the nonmagnetic metal $PdCoO_2$. *Phys. Rev. Lett.* **111,** 056601 (2013).

3. Moll, P. J. W. *et al.* Evidence for hydrodynamic electron flow in $PdCoO_2$. *Science* **351,** 1061-1064 (2016).
